# Great Northern Central Hospital

**Published:** 1894-11-24

**Authors:** Robert Burnet, J. Macready

**Affiliations:** Senior Physician. Great Northern Central Hospital; Senior Surgeon. Great Northern Central Hospital


### EDITOR'S LETTER-BOX.

[Our correspondents are reminded that prolixity is a great bar to publication, and that brevity of style and conciseness of statement greatly facilitate early insertion.]

### GREAT NORTHERN CENTRAL HOSPITAL.

We have received the subjoined copy of a letter addressed on behalf of the medical staff of this hospital to the chairman of a committee appointed by 201 medical practitioners in North London:—

Dear Sir,—We desire to acknowledge on the part of our colleagues your courtesy in submitting to the medical staff of the hospital a copy of the protest recently addressed to the Committee of Management. The reply given by the chairman of that committee to the deputation on October 19th ultimo will already have made it clear that some sections of the protest were drawn up without a full knowledge of the circumstances attending the establishment of pay wards at the Great Northern Central Hospital. In order that no misapprehension may remain as to the facts of the case, we have been asked by our colleagues to explain them once more, and especially from the point of view of the medical staff. It must be clearly understood that the demand for pay wards came entirely from the supporters of a proposed new hospital in North London in the year 1883, and that the idea was warmly advocated at that time by several medical practitioners in the district. It was one of the conditions of the establishment of the hospital, that there should be pay wards in the future, and the then medical staff of the old Great Northern Hospital in the Caledonian Road was appointed to the new hospital on that understanding. But this innovation was considered so hazardous that a special meeting of the staff was at once called, and a definite resolution was passed to the effect that the medical staff would refuse to have anything to do with any scheme that might be thereafter proposed which should involve the making of a pecuniary profit by the hospital. It has been frequently stated at meetings of the governors and subscribers and duly reported in the Press, that these pay wards were intended for the reception of patients a little above the poorer class, in fact, for the very class of patients who are so constantly recommended by medical men for admission into the free wards, although able to contribute towards their maintenance. So long as admission to the pay wards was to be restricted to such cases, the present staff felt that they could not refuse their services, but they stipulated for and obtained the following conditions: (1) That no patient should be admitted without the consent, in writing, of his or her own medical attendant. (2) That the medical staff should have control with the House Committee of the admission of such patients. With these safeguards it was hoped that abuse of the pay wards would be almost impossible, and already a large proportion of the applications have been rejected. It will thus be seen that this system differs entirely from any others which have been hitherto tried. The staff, when asked by the Committee of Management to frame rules for the proposed scheme, was opposed to the adoption of any of the existing methods of working pay wards. They endeavoured, therefore, to devise a new plan, which might, as nearly as possible, fulfil the intentions expressed by the governors in 1883, and which should be without prejudice to the interests of the profession or of the nursing homes in the district. The working of this experiment will be closely watched, and prompt action will be taken by the staff if it can be shown that these interests are endangered. The abolition of the whole system, asked for by the deputation, can only be effected by those [illegible] instituted it, viz., the professional and lay governors of the hospital.—We remain, dear sir, yours faithfully, (Signed on behalf of the whole staff)

Robert W. Burnet, Senior Physician.
J. Macready, Senior Surgeon.

Great Northern Central Hospital, November 20th, 1894.

